# Protective Effects of Edaravone in Adult Rats with Surgery and Lipopolysaccharide Administration-Induced Cognitive Function Impairment

**DOI:** 10.1371/journal.pone.0153708

**Published:** 2016-04-26

**Authors:** Peiqi Wang, Jiangbei Cao, Na Liu, Li Ma, Xueyue Zhou, Hong Zhang, Yongan Wang

**Affiliations:** 1 Department of Anesthesiology and Operation Center, Chinese PLA, General Hospital, Beijing, China; 2 Department of Anesthesiology, Affiliated Hospital of Chengde Medical University, Chengde, China; 3 Department of Anesthesiology, Beijing Military General Hospital of the Chinese People’s Liberation Army, Beijing, China; 4 State Key Laboratory of Toxicology and Medical Countermeasures, Academy of Military Medical Sciences, Beijing, China; Massachusetts General Hospital, UNITED STATES

## Abstract

Postoperative cognitive dysfunction (POCD) is a clinical syndrome characterized by cognitive declines in patients after surgery. Previous studies have suggested that surgery contributed to such impairment. It has been proven that neuroinflammation may exacerbate surgery-induced cognitive impairment in aged rats. The free radical scavenger edaravone has high blood brain barrier permeability, and was demonstrated to effectively remove free radicals from the brain and alleviate the development of POCD in patients undergoing carotid endarterectomy, suggesting its potential role in preventing POCD. For this reason, this study was designed to determine whether edaravone is protective against POCD through its inhibitory effects on inflammatory cytokines and oxidative stress. First, Sprague Dawley adult male rats were administered 3 mg/kg edaravone intraperitoneally after undergoing a unilateral nephrectomy combined with lipopolysaccharide injection. Second, behavioral parameters related to cognitive function were recorded by fear conditioning and Morris Water Maze tests. Last, superoxide dismutase activities and malondialdehyde levels were measured in the hippocampi and prefrontal cortex on postoperative days 3 and 7, and microglial (Iba1) activation, p-Akt and p-mTOR protein expression, and synaptic function (synapsin 1) were also examined 3 and 7 days after surgery. Rats that underwent surgery plus lipopolysaccharide administration showed significant impairments in spatial and working memory, accompanied by significant reductions in hippocampal-dependent and independent fear responses. All impairments were attenuated by treatment with edaravone. Moreover, an abnormal decrease in superoxide dismutase activation, abnormal increase in malondialdehyde levels, significant increase in microglial reactivity, downregulation of p-Akt and p-mTOR protein expression, and a statistically significant decrease in synapsin-1 were observed in the hippocampi and prefrontal cortices of rats at different time points after surgery. All mentioned abnormal changes were totally or partially reversed by edaravone. To our knowledge, few reports have shown greater protective effects of edaravone on POCD induced by surgery plus lipopolysaccharide administration from its anti-oxidative stress and anti-inflammatory effects, as well as maintenance of Akt/mTOR signal pathway activation; these might be closely related to the therapeutic effects of edaravone. Our research demonstrates the potential use of edaravone in the treatment of POCD.

## 1. Introduction

Postoperative cognitive dysfunction (POCD) refers to varying degrees of cognitive function decline in patients after surgery. It covers a wide range of cognitive functions including working memory, long term memory, information processing, attention, and cognitive flexibility [1, 2]. POCD adversely affects quality of life, social dependence, and mortality [3]. Oxidative stress, surgery, general anesthesia/anesthetics, and neuroinflammation are believed to increase the risk of POCD [4–6].

Certain tissues can be damaged as a result of oxidative stress, especially during an operation [7]. The free radical scavenger edaravone, which crosses the blood brain barrier, can effectively remove free radicals from the brain [8]. Evidence has shown that oxidative factors were harmful to cognitive function [9–10]. However, edaravone can improve the cholinergic system and protect neurons from oxidative toxicity, alleviate Alzheimer’s disease-type pathologies, and cognitive deficits [11, 12]. Other studies demonstrated that edaravone inhibited the progression of cerebral infarction and ischemia [13, 14]. Most importantly, the effects of edaravone on the development of POCD have been proven in patients undergoing carotid endarterectomy[15] In short, previous studies suggest that edaravone might improve cognitive impairment in patients after surgery by scavenging free radicals.

Lipopolysaccharide (LPS) is a major bacterial TLR4 ligand that activates the immune response to infections [16]. Recent reports have demonstrated that surgery accompanied by LPS treatment triggered more severe neurodegeneration in adult rats [17]; The interaction between oxygen free radicals and inflammatory factors would exacerbate postoperative cognitive dysfunction[18,19].They both would destroy cell membrane function, break the balance of homeostasis, cause oxidative phosphorylation in a mess[20]. The normal activation of the Akt/mTOR signal pathway was the phosphorylation[21]. a subsequent increase in activated microglial cells and inhibition of activation of the Akt/mTOR signal pathway were also observed, finally leading to declines in learning and memory [22, 23]. Also, mTOR was involved in regulating synaptic plasticity, which affected the function of memory and cognitive [24,25]. Based on previous reports, we hypothesized that in a rat model of surgery associated with LPS administration, edaravone might improve POCD by alleviating oxidative toxicity, inhibiting microglial activation, and maintaining normal function of activation of the Akt/mTOR signal pathway. The results obtained in this study may provide new insights into the potential roles of edaravone in the treatment of POCD, as well as its mechanisms of action.

## 2. Materials and Methods

### 2.1 Animals

Adult male Sprague Dawley rats (n = 80) aged 8 weeks and weighing 220–250 g were purchased from Vital River Laboratories Animal Technology Co. Ltd. (Beijing, China. Permit Number: SCXK (JING) 2012–0001). All rats were housed under controlled conditions with a 12-h light/dark cycle and *ad libitum* access to food and water for 7 days before the experiment. The procedures on animal experimentation were approved by the Animal Care Committee of the Chinese People’s Liberation Army General Hospital (Beijing, China). The maintenance and handling of the rats were consistent with the guidelines of the National Institutes of Health, and adequate measures were taken to minimize animal discomfort. The rats were divided into four groups randomly (20 rats per group): the control plus placebo group (C-P), control plus edaravone group (C-E), surgery plus placebo group (S-P), and surgery plus edaravone group (S-E). Each group was divided into two subgroups randomly (10 rats per group): the 3-day postoperative group and 7-day postoperative group. The C-P and S-P groups received a placebo (0.3 mL of saline by intraperitoneal [i.p.] injection), and the C-E and S-E groups received 3 mg/kg of edaravone (Cat: 80–131003, Simcere, Nanjing, China) in 0.3 mL of saline by i.p. injection.

### 2.2 Surgical Procedures

**Fig 1:**
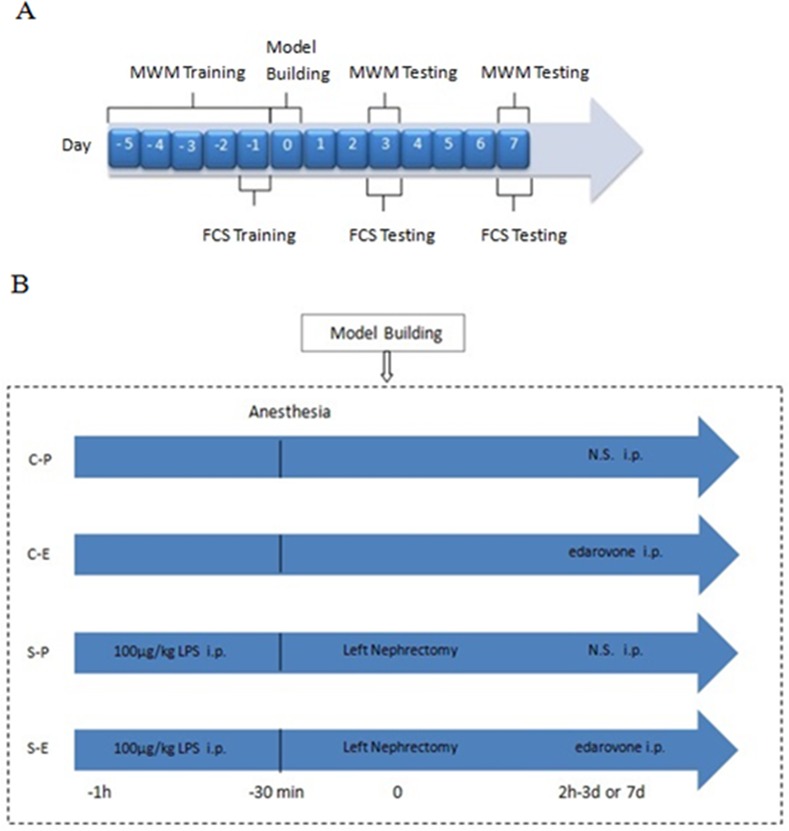
The schematic outline of the experimental protocol and the timeline of LPS and edaravone administration. (A)Schematic outline of the experimental protocol. (B) Timeline of LPS and edaravone administration. MWM, Morris water maze; FCS, Fear conditioning test;C-P, sham surgery plus placebo; C-E, sham surgery plus edaravone; S-P, surgery plus placebo; S-E, surgery plus edaravone.

After undergoing the Morris Water Maze (MWM) test and fear conditioning training for 5 consecutive days, animals in the S-P and S-E groups underwent LPS administration of 100 μg/kg i.p. (Sigma, St. Louis, MO, USA). The dosage of LPS was determined according to a previous report [17]. After 1 h, the LPS-treated groups underwent a left nephrectomy under pentobarbital sodium anesthesia (1% and 40 mg/kg) (Fig 1A). A longitudinal incision was made in the back where the wounds were not accessible to the rats to avoid self-inflicted bite trauma. We considered this surgery model to mimic a standardized organ removal in humans with sub-clinical infection [8]. During the operation, the rats’ body temperature were maintained at 36.5°C to 37.5°C. All rats received 50μl of 0.2% ropivacaine subcutaneously for the post-operative analgesia. Rats were allowed to recover in an incubator at 37°C and were then returned to their cages. Later, the C-P and S-P groups received saline (i.p.), whereas the C-E and S-E groups received edaravone (i.p.) each day until days 3 and 7 after surgery, respectively(Fig 1B). Each rat would be weighted every day after operation, all rats were put on weight with days. We sterilized the wound of rats at the day 1,2,3,5,7 after surgery. Then, the animals were sacrificed with the lethal dose of pentobarbital sodium i.p. at 3 days and 7 days postoperatively, and the brains of five rats in each group were immediately removed and fixed in 4% paraformaldehyde for 48 h for histological analysis. The hippocampi and prefrontal cortices of the other rats were rapidly dissected, removed, and stored at -80°C until analysis.

### 2.3 Behavior tests

#### 2.3.1 MWM test

The MWM test (EthoVision, The Netherlands) was performed to assess spatial learning, spatial memory, and cognitive flexibility in the rats [26]. The water maze consisted of a round container (180 cm × 60 cm) made of black plastic and filled with water (25 ± 1°C). The pool was placed in a room with several visual cues for orientation in the maze. The maze was divided into four quadrants: the first, second, third, and fourth quadrants. An invisible platform (10 cm × 10 cm) was placed 1 cm below the water surface in the first quadrant (target quadrant). All rats underwent repeated training for 5 consecutive days. Every day, they were released successively into the water facing the wall of the pool from the first quadrant to the fourth quadrant. The rats were trained to find the hidden platform and climb onto it within 60 s. The animals were allowed to stay on the platform for at least 10 s after each trial. When the rats failed to reach the escape platform within 60 s, they were gently guided towards the platform and left there for 10 s. After the completion of four trials, the rat was dried with a towel and returned to its cage. The animals’ movements were recorded with a video camera.

On postoperative day 3, probe tests were conducted on all the treated groups by removing the platform and releasing the rats in the third quadrant (opposite to the first quadrant). Latency, the number of crossings over the former location of the platform, and time spent in each quadrant were measured in a single 60-s trial. Then, working memory was tested; both the platform and rat were randomly placed in novel positions to assess trial-dependent learning and working memory [27]. Animals underwent one more training session to ensure that all rats learned the new platform location. After 15 s, each rat was released from the same location as in the above training; the rat would swim a shorter path to the platform in the second trial if it recalled the first trial. The escape latency to the platform in the second trial was taken as measure of temporary or working memory. All of the 7-day postoperative groups underwent the same trials on postoperative day 7.

#### 2.3.2 Fear conditioning

Fear conditioning is used to detect associative learning and memory function [28]. Different groups of rats were trained for fear conditioning 1 day before the operation. Rats were subjected to an inescapable electric foot shock provided via the grid floor of a testing chamber. The chamber in which training occurred was lit with fluorescent bulbs. The total training time was 330 s for each rat. Each animal was allowed to explore the chamber for 60 s before the presentation of 3 tone-foot shock pairings (tone: 2000 Hz, 85 dB, 30 s; foot shock: 0.9 mA, 2 s) with an intertribal interval of 60 s. Then, the animal was removed from the test chamber 60 s after conditioning training.

Different groups underwent the context test and tone test on postoperative days 3 and 7, respectively. The rats were tested in the context and tone test. Each animal was placed into the chamber for 330 s either in a context test (without a tone or shock) or a tone test (without a shock). Episodes of freezing were recorded by a digital camera. These tests assessed hippocampi-dependent (context-related) and hippocampi-independent (tone-related) learning and memory functions [29]. They were expressed as the percentage of freezing time using software analysis.

### 2.4 Biochemical analysis

#### 2.4.1 Malondialdehyde (MDA)

MDA is one of the lipid peroxides. The concentration of MDA indicates how severely tissue is attacked by free radicals. This method is based on thibabituric acid (TBA). The color reaction was measured at 532 nm. The levels of MDA in the hippocampi and prefrontal cortices of rats were measured using commercial assay kits (Nanjing Jiancheng Bioengineering Institute, Nanjing, China) according to the manufacturer’s instructions.

#### 2.4.2 Superoxide dismutase (SOD) activity

The method was based on the ability of SOD to inhibit the superoxide anion free radical O_2_^-^. The color reaction was measured at 550 nm. The SOD activity of tissue was also measured using commercial assay kits (Nanjing Jiancheng Bioengineering Institute, Nanjing, China).

### 2.5 Immunofluorescence staining

A cerebral block containing the hippocampi and prefrontal cortex was fixed in 10% neutral-buffered formalin overnight and then embedded in paraffin. Coronal 10-μm sections were prepared and subjected to immunofluorescence staining. First, paraffin sections were dewaxed and placed in EDTA buffer (pH 8.0) to repair antigens. Second, sections were washed in 0.01% Triton X-100 in phosphate-buffered saline (PBS-T) and blocked with 3% bovine serum albumin (BSA) for 30 min at room temperature. Then, they were incubated overnight at 4°C in appropriate primary antibodies: anti-Iba1 (1:100; WAKO) and anti-synapsin-1 (1:100; Cell Signaling). Next, the sections were incubated with the appropriate secondary antibodies including anti-rabbit IgG (1:400; Jackson) and anti-mouse IgG (1:400, Jackson) for 2 h at room temperature. The number of positively stained microglial cells was counted by fluorescence microscopy at 400× magnification and the mean density of the synapses was also calculated by fluorescence microscopy at 400× magnification.

### 2.6 Western blot

The hippocampal and prefrontal cortical tissues were homogenated in RIPA buffer (50 mmol/L Tris–HCl, pH 6.8, 150 mmol/L NaCl, 5 mmol/L EDTA, 0.5% sodium deoxycholate, 0.5% NP-40, and supplemented with a cocktail containing protease and phosphatase inhibitors). The total lysates were centrifuged at 12000 rpm for 30 min at 4°C. Protein concentrations were determined by a BCA Protein Assay reagent kit (Pierce, Rockford, IL, USA). Equal amounts of the sample (30 μg of protein) were separated by SDS-PAGE and analyzed by Western blot using the following primary antibodies: rabbit polyclonal anti-Akt and anti-p-Akt (1:1000, Cell Signaling), rabbit polyclonal anti-p-mTOR (1:1000, Cell Signaling), and mouse monoclonal anti-β-actin polyclonal antibody (1:3000; Abcam). Appropriate secondary antibodies were used. Each experiment was repeated no less than four times. Relative expression was normalized to β-actin.

### 2.7 Statistical analysis

All data were analyzed by an observer who was blinded to the experimental protocol. Statistical calculations were performed using SPSS 16.0 (SPSS Science, Inc., Chicago, IL, USA). We analyzed multiple group means by a two-way analysis of variance followed by Dunnett’s *post hoc* test wherever appropriate. Values of *p* < 0.05 were considered significant.

## 3. Results

### 3.1 Edaravone attenuated unilateral nephrectomy plus LPS administration-induced learning and memory impairment

**Fig 2:**
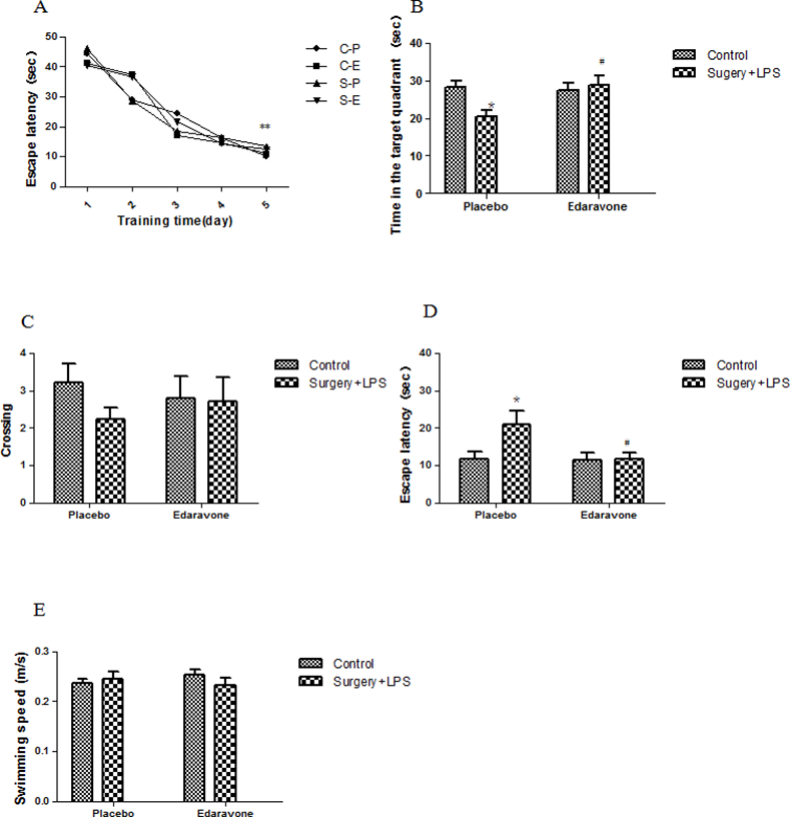
**Edaravone attenuated behavioral performance after unilateral nephrectomy plus LPS administration in rats** (A) Spatial learning in the MWM. Average escape latency (s) is shown for the five training sessions in the maze. (B) Dwelling time in the target quadrant in the first MWM probe trial on day 3 after surgery. (C) The number of crossings on postoperative day 3. (D) Average escape latency (s) during the MWM reversal trials on day 3 after surgery. (E) Average swimming speed (m/s) in the first MWM probe trial. **P< 0.01 vs. the first day since training; *P< 0.05 vs. C-P group; #*P* <0.05 vs. S-P group. C-P, sham surgery plus placebo; C-E, sham surgery plus edaravone; S-P, surgery plus placebo; S-E, surgery plus edaravone.

Previous work has demonstrated that a nephrectomy plus an LPS injection could lead to POCD [17]. Therefore, the protective effects of edaravone on POCD were examined in this model. As shown in Fig 2A, in the MWM test, the escape latency in all groups was significantly shorter during the last training session when compared to the first training session (*p* < 0.001), yet no difference was observed between the groups, indicating that all animals were able to learn where the platform was located. On postoperative day 3, the well time in the target quadrant in the first MWM probe trial in the S-P group was decreased notably compared to the other groups (*p <* 0.05), and the number of crossings also showed a decreasing tendency, although it did not reach significance (Fig 2B and 2C). In the working memory test, the escape latency needed to reach the new platform was increased obviously (*p <* 0.05) in the S-P group compared to the C-P and S-E groups (Fig 2D). During the probe test, there were no significant difference in swimming speed between the groups, suggesting that the poorer performance of the S-P group was not a result of reduced motor ability (Fig 2E). On postoperative day 7, there was no statistical difference between the S-P group and other groups in dwelling time in the target quadrant, number of crossings, or escape latency, although rats in the S-P group presented a decreasing tendency in dwelling time in the target quadrant and an increasing tendency in escape latency.

**Fig 3:**
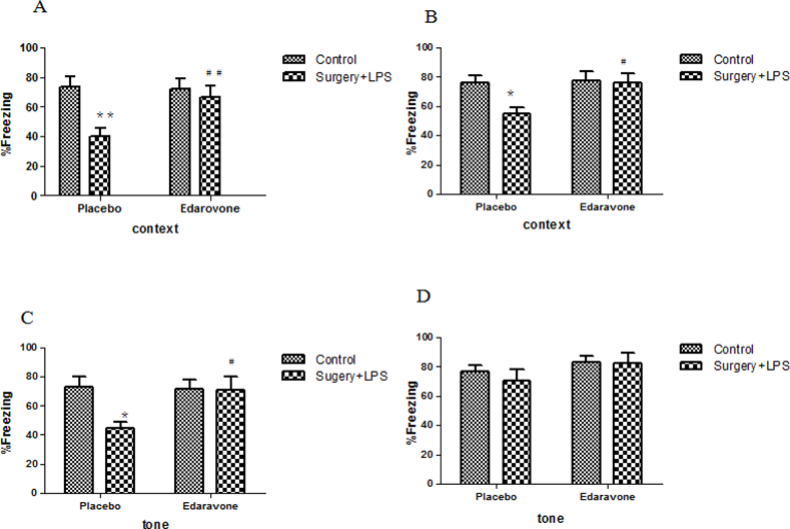
**Cognitive impairment after surgery in the fear conditioning test** (A) The hippocampal-dependent memory test on day 3 after surgery. (B) The hippocampal-dependent memory test on day 7 after surgery. (C) The hippocampal-independent memory test on postoperative day 3. (D) The hippocampal-independent memory test on postoperative day 7. *P< 0.05, **P< 0.01 vs. C-P group; #*P* <0.05, ##*P* <0.01 vs. S-P group. C-P, sham surgery plus placebo; C-E, sham surgery plus edaravone; S-P, surgery plus placebo; S-E, surgery plus edaravone.

In the fear conditioning test, hippocampal-dependent memory was assessed in a novel context and revealed highly significant impairment in the S-P group when compared to the C-P group on postoperative days 3 (*p* < 0.01) and 7 (*p <* 0.05) (Fig 3A and 3B). Compared to the C-P group, the freezing time in the S-P group was significantly decreased (*p* < 0.01). This decrease was reversed obviously in the S-E group (*p* < 0.05/0.01), indicating the protective effects of edaravone on the development of POCD. During the tone-related fear conditioning test (hippocampal-independent memory) on postoperative day 3, as shown in Fig 3C, the freezing time percentage was notably decreased in the S-P group when compared to the C-P group (*p* < 0.05); this decrease was significantly prevented by edaravone (*p* < 0.05). On postoperative day 7, freezing responses to the tone were not significantly different between any of the groups (Fig 3D).

### 3.2 Edaravone increased SOD activities and reduced MDA levels in the hippocampi and prefrontal cortex in rats after surgery plus LPS administration

**Fig 4:**
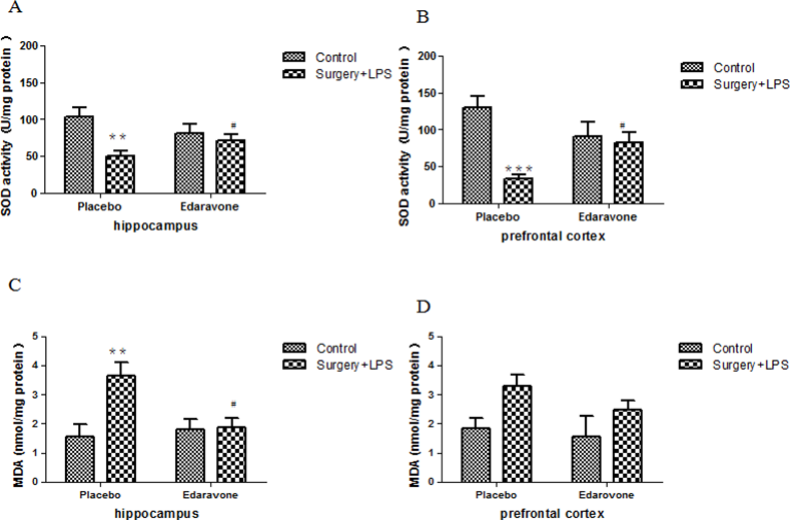
**Edaravone increased SOD activities and reduced hippocampal and prefrontal cortex MDA levels after surgery** (A) SOD activities in the hippocampi on postoperative day 3. (B) SOD activities in the prefrontal cortex on postoperative day 3. (C) The MDA level in the hippocampi on postoperative day 3. (D) The MDA level in the prefrontal cortex on postoperative day 3. *P< 0.05, **P< 0.01, ***P< 0.001 vs. C-P group; #*P* <0.05 vs. S-P group. C-P, sham surgery plus placebo; C-E, sham surgery plus edaravone; S-P, surgery plus placebo; S-E, surgery plus edaravone.

As demonstrated in Fig 4A and 4B, compared to the C-P group, the SOD activities of the hippocampi and prefrontal cortex were significantly decreased on postoperative day 3 (*p* < 0.01/0.001), but showed no change on postoperative day 7 in the S-P group; this abnormal decrease in SOD activities was largely prevented by edaravone (*p <* 0.05). Likewise, edaravone significantly attenuated abnormally increased MDA levels in the hippocampi of the S-P group 3 days after the operation (*p* < 0.01) (Fig 4C). No difference was observed between the groups regarding MDA level in the prefrontal cortex on postoperative day 3 (Fig 4D) or day 7, although the MDA level in the S-P group also showed an increasing tendency without a statistical difference.

### 3.3 Edaravone prevented microglial activation after surgery plus LPS administration

**Fig 5:**
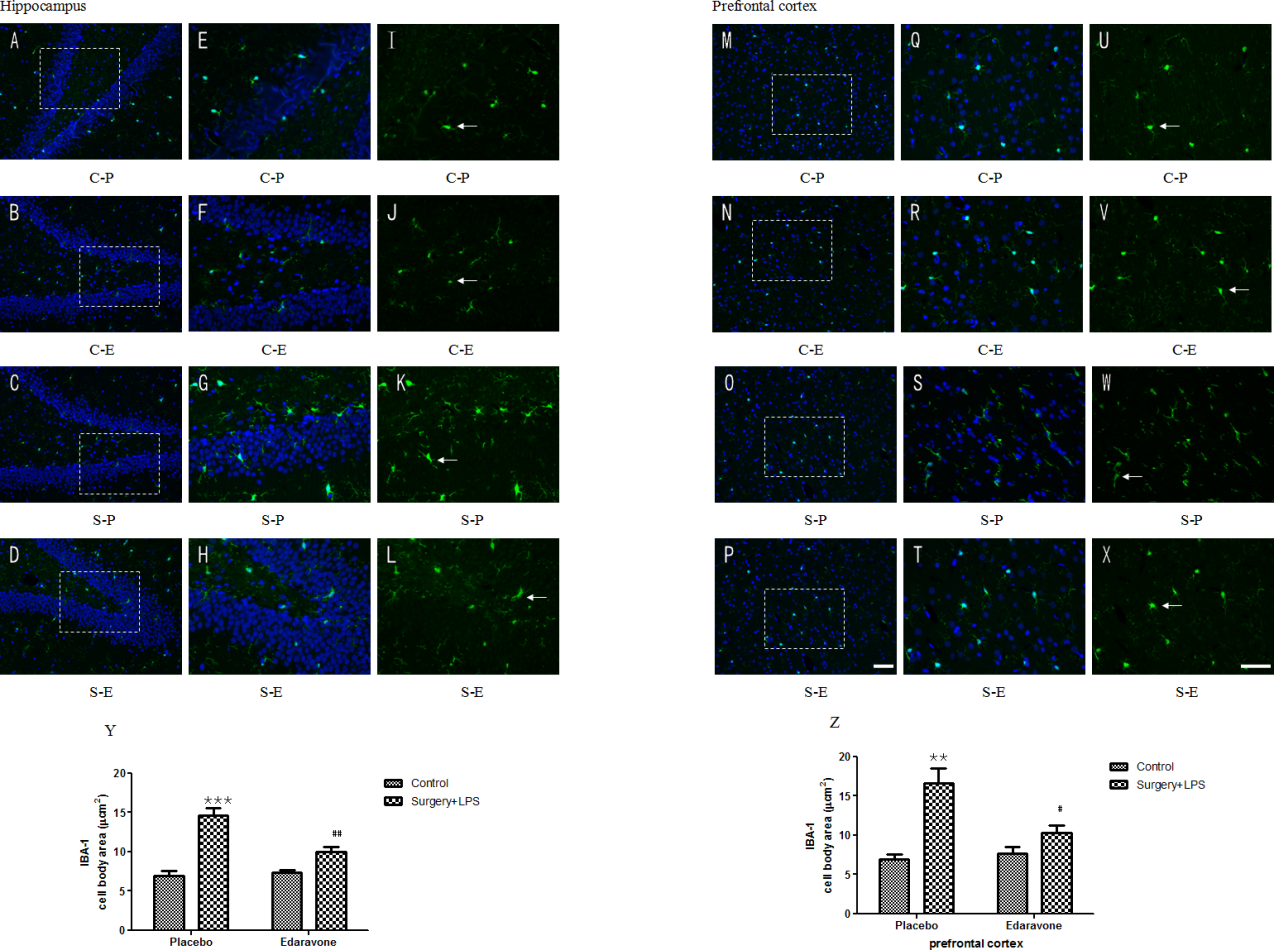
**Edaravone attenuated surgery plus LPS-induced microglial (green) activation** (A-X) Representative images of Iba1-labeled activated microglia in the hippocampi and prefrontal cortex. (A-D) Activated microglia and cell nuclei in the hippocampi on postoperative day 3 under a 200× magnification fluorescence microscope. (E-H) Activated microglia and cell nuclei in the hippocampi on postoperative day 3 under a 400× magnification fluorescence microscope. (I-L) Activated microglia in the hippocampi on postoperative day 3 under a 400× magnification fluorescence microscope. (M-P) Activated microglia and cell nuclei in the prefrontal cortex on postoperative day 3 under a 200× magnification fluorescence microscope. (Q-T) Activated microglia and cell nuclei in the prefrontal cortex on postoperative day 3 under a 400× magnification fluorescence microscope. (U-X) Activated microglia in the prefrontal cortex on postoperative day 3 under a 400× magnification fluorescence microscope. (Y) The number of hippocampal Iba1-positive cells on postoperative day 3. (Z) The number of prefrontal cortical Iba1-positive cells on postoperative day 3. Scale bars: A-D and M-P, 100 μm; E-L and Q-X, 50 μm. *P< 0.05, **P< 0.01, ***P< 0.001 vs. C-P group; #*P* <0.05, ##*P* <0.01 vs. S-P group. C-P, sham surgery plus placebo; C-E, sham surgery plus edaravone; S-P, surgery plus placebo; S-E, surgery plus edaravone.

Using immunofluorescence, the effects of edaravone on ionized calcium binding adapter molecule 1 (Iba1) were investigated. As shown in Fig 5A–5X, the total counted number of Iba1-positive cells on hippocampal (Fig 5Y; *p* < 0.001) and prefrontal cortical (Fig 5Z; *p* < 0.01) sections in the S-P group was much higher than in the C-P group and S-E group on postoperative day 3, yet there were no significant difference among the treated groups on postoperative day 7.

### 3.4 Edaravone attenuated surgery plus LPS administration-induced neuroinflammation

**Fig 6:**
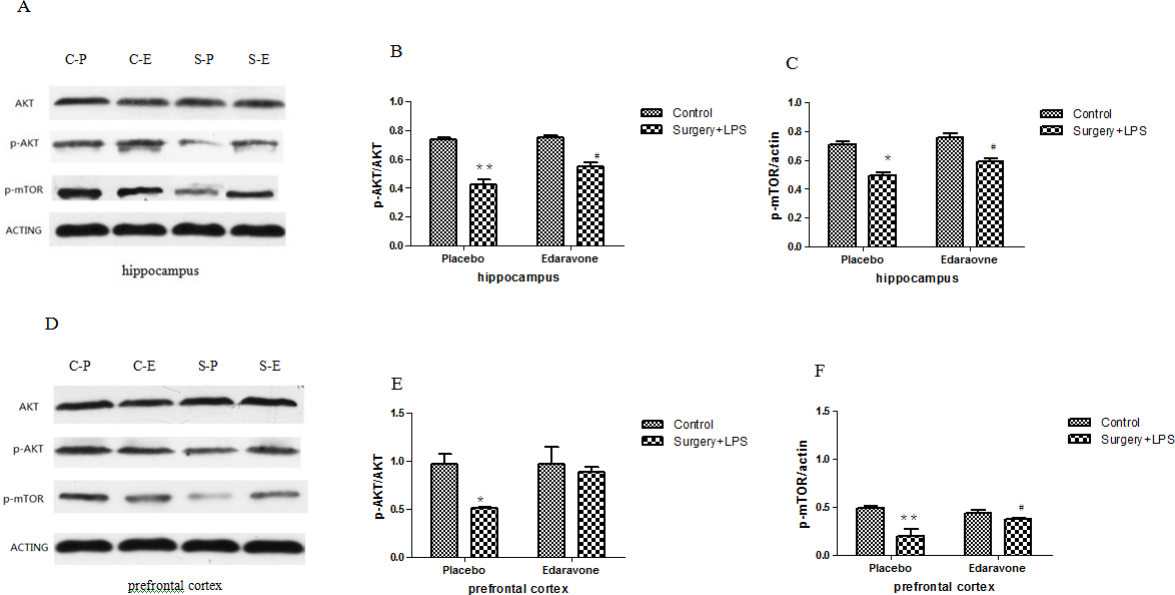
**Effects of edaravone on protein expression in rats with LPS-induced hippocampal and prefrontal cortical impairment** (A) The expression of related protein in the hippocampi on day 3 after surgery. (B) The ratio of p-Akt/Akt in the hippocampi on day 3 after surgery. (C) The ratio of p-mTOR/actin in the hippocampi on day 3 after surgery. (D) The expression of related protein in the prefrontal cortex on postoperative day 3. (E) The ratio of p-Akt/Akt in the prefrontal cortex on postoperative day 3. (F) The ratio of p-mTOR/actin in the prefrontal cortex on postoperative day 3. *P< 0.05, **P< 0.01 vs. C-P group; #*P* <0.05 vs. S-P group. C-P, sham surgery plus placebo; C-E, sham surgery plus edaravone; S-P, surgery plus placebo; S-E, surgery plus edaravone.

To further investigate the mechanism of edaravone in preventing microglial activation, Akt/mTOR signal pathway-related protein expression was tested by western blot. As shown in Fig 6A–6F, on day 3 after the operation, protein levels of p-Akt and p-mTOR in the rats’ hippocampi and prefrontal cortices were largely decreased in the S-P group compared to the C-P group (*p* < 0.05/0.01); this abnormal decrease was significantly attenuated (*p* < 0.05) by edaravone. On postoperative day 7, no difference in protein expression was observed between any of the groups.

### 3.5 Edaravone improved surgery plus LPS administration-induced synaptic function depression

**Fig 7:**
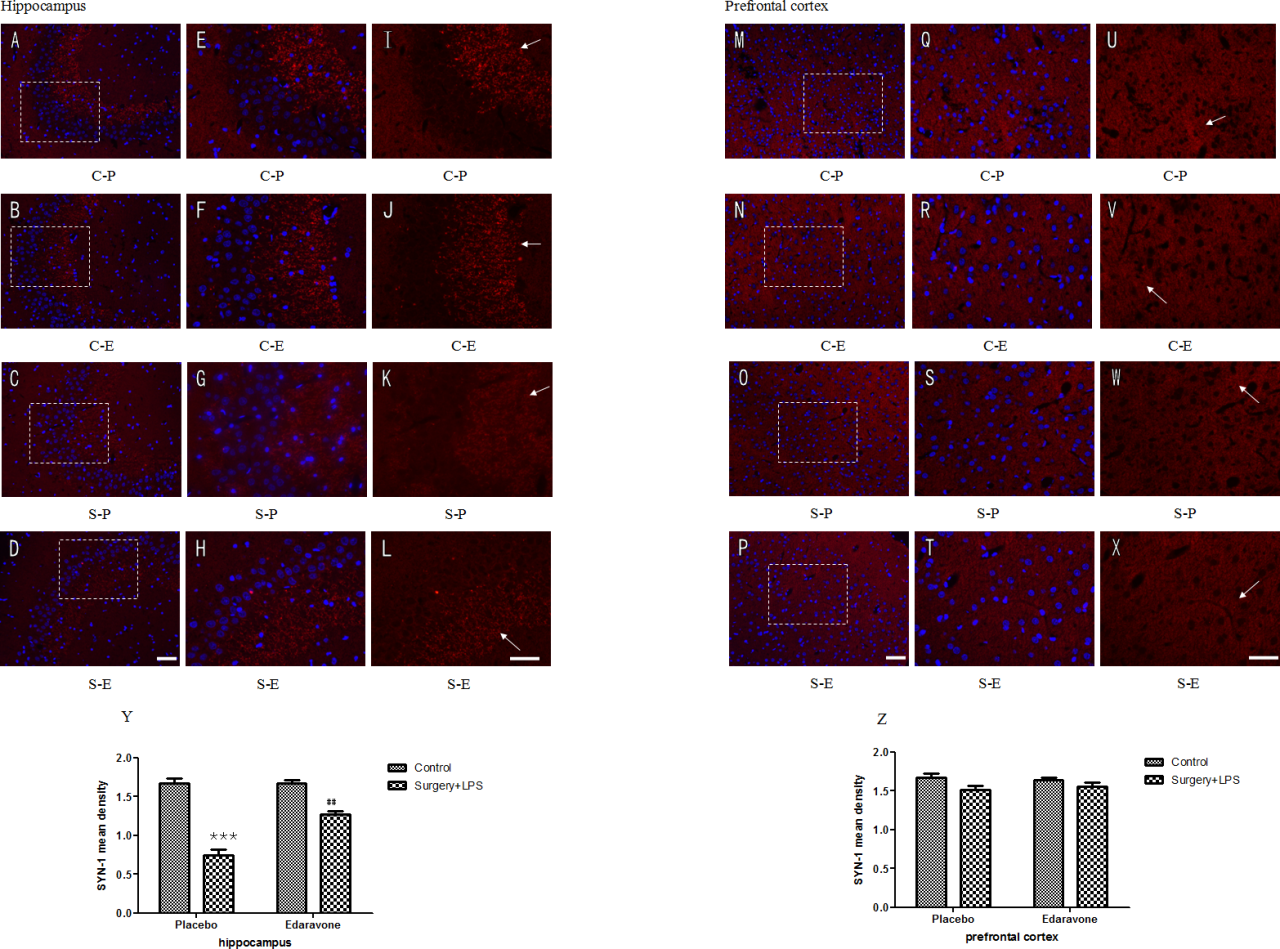
**Edaravone protected hippocampal and prefrontal cortical synaptic (red) integrity after surgery plus LPS administration** (A-L) Representative images of SYN-labeled synapses in the hippocampi. (A-D) Synaptic protein and cell nuclei in the hippocampi on postoperative day 3 under a 200× magnification fluorescence microscope. (E-H) Synaptic protein and cell nuclei in the hippocampi on postoperative day 3 under a 400× magnification fluorescence microscope. (I-L) Synaptic protein in the hippocampi on postoperative day 3 under a 400× magnification fluorescence microscope. (M-P) Synaptic protein and cell nuclei in the prefrontal cortex on postoperative day 3 under a 200× magnification fluorescence microscope. (Q-T) Synaptic protein and cell nuclei in the prefrontal cortex on postoperative day 3 under a 400× magnification fluorescence microscope. (U-X) Synaptic protein in the prefrontal cortex on postoperative day 3 under a 400× magnification fluorescence microscope (Y) The density of hippocampal synaptic protein on postoperative day 3. (Z) The density of prefrontal cortical synaptic protein on postoperative day 3. Scale bars: A-D, 100 μm; E-L, 50 μm. ***P< 0.001 vs. C-P group; ##*P* <0.01 vs. S-P group. C-P, sham surgery plus placebo; C-E, sham surgery plus edaravone; S-P, surgery plus placebo; S-E, surgery plus edaravone.

To further evaluate the protective effects of edaravone on surgery plus LPS administration-induced cognitive function impairment, the synaptic protein SYN was examined. On postoperative day 3, a significant reduction in SYN intensity was observed in hippocampi from group S-P (*p* < 0.001) (Fig 7A–7L), and this reduction was partially reversed (*p* < 0.01) by edaravone(Fig 7Y). On postoperative day 7, the SYN intensities in the hippocampi showed no difference. Different from the hippocampi, the expression of SYN in the prefrontal cortex was not different between any of the groups on postoperative day 3 (Fig 7M–7X and 7Z) or day 7.

## 4. Discussion

This paper shows that surgery plus LPS injection can induce POCD in rats, and that the resulting cognitive impairment can be largely prevented by edaravone. Moreover, the protective effects of edaravone on the development of POCD in rats may be related to its antioxidant effects, inhibiting microglial activation, and maintaining normal activation of the Akt/mTOR signal pathway.

Recent studies revealed that surgery can lead to cognitive decline by triggering systemic and hippocampal inflammation [5, 30, 31]. Systemic infection increases the levels of pro-inflammatory cytokines in the brain that contribute to subsequent impairment of the consolidation of memory in rats [32]. LPS, the major component of the outer membrane of Gram-negative bacteria, is known to trigger a powerful immune response [16]. Priming the immune system with a subclinical dose of LPS can amplify the pro-inflammatory response caused by surgery [33]. In clinical practice, it is very common for patients to have sub-clinical infection before or after an operation [17]. For this reason, based on the reported studies, we chose the dosage of LPS (100 μg/kg) to mimic sub-clinical infection. The chosen dose has been tested and has the ability to sensitize the immune system and augment the severity of unilateral nephrectomy-induced impairment of cognition [17].

The MWM test was chosen as a robust and reliable test that is strongly correlated with hippocampal-dependent memory [34–35]. It consists of two parts: the spatial reference memory test and reversal test. In the spatial reference memory test, obviously inherent memory impairment was observed in the S-P group, and this inherent memory injury was significantly alleviated by edaravone. In the MWM reversal task, a method was used to evaluate cognitive flexibility, which is independent of hippocampal function [36]. The obvious reduction in learning ability and short-term memory were shown in the S-P group, and this cognitive impairment after the operation was also prevented by edaravone. In the novel context test of fear conditioning, hippocampal-dependent cognitive dysfunction was sustained on postoperative day 7, whereas hippocampal-independent cognitive decline occurred after postoperative day 3, but did not last to postoperative day 7. Edaravone administration also prevented cognitive decline and accelerated cognitive recovery in the fear conditioning test.

In the context fear conditioning test, the cognitive dysfunction was sustained on postoperative day 7, while the spatial reference memory in the MWM test on postoperative day 7 was not changed in surgery plus LPS group. It maybe related to rats form different memory with different regions of hippocampi, and the damage degree of hippocampal regions which surgery plus LPS induced was different. Although spatial memory and contextual fear memory were hippocampal-dependent, the formation of memory depended on different brain regions[37].The spatial memory rely on hippocampi, corpus striatum, basal forebrain, cerebellum and other regions participation, any damage of above tissues will induce memory impairment[38]. The impairment of dorsal hippocampi was more serious than the impairment of ventral hippocampi for spatial memory decline[39].Fear conditioning test formed cortex memory, it relied mainly on CA1 region of hippocampi[40]. Especially, the activity of RA1 was associated with the fear cortex memory[41].

Previous studies have shown that cognitive impairment was obvious in water maze and fear conditioning tests in unilateral nephrectomy-treated aged rodents [42, 43]. Meanwhile, systemic inflammation is believed to increase the levels of pro-inflammatory cytokines in the brain and aggravate POCD [17, 32]. Edaravone, a known antioxidant, has been demonstrated to antagonize POCD in patients [15]. However, to our knowledge, few studies have examined the protective effect of edaravone in POCD induced by surgery plus LPS injection. Our study is the first to demonstrate the potential role of edaravone in the treatment of cognitive impairment caused by surgery plus LPS injection.

Previous studies have indicated that surgery contributed to the inflammatory response and oxidative stress by activating the immune system [44,45], and systemic infection would result in more inflammatory cytokines in the brain [32]. Both inflammation and oxygen free radicals were believed to take part in the onset and maintenance of POCD [46, 47]. Moreover, inflammation also promoted the entrance of oxygen free radicals into the central nervous system [29] and then exacerbated the injurious effects of oxidative stress on cognitive function [18]. For these reasons, the antioxidant and anti-neuroinflammation effects of edaravone were further investigated in rats that underwent surgery plus an LPS injection.

Abnormal changes in the activities of SOD and the levels of MDA in brain tissues were thought to relate to dysfunction and damage to the structure of the cell membranes, mitochondria, and lysosomes, as well as cell autolysis related to POCD [46]. In addition, the overexpression of inflammatory cytokines was often accompanied by an increased number of activated microglial cells [48, 49], which were characterized by an acute increase in Iba1. In this paper, decreased activities of SOD and increased levels of MDA, as well as a significant increase in Iba1, were shown at different time points after the operation (days 3 and 7 for SOD and MDA, and day 3 for Iba1) in the hippocampi and prefrontal cortices of S-P group animals. All the above-mentioned abnormal changes were partially reversed by edaravone, further suggesting that the protective effects of edaravone on POCD might be related to its antioxidant and anti-neuroinflammation effects.

In addition to attenuating oxidative stress and neuroinflammation, maintaining the activation of the Akt/mTOR signal pathway to prevent POCD induced by surgery by inhibiting inflammation was thought to be a reliable method [50]. The reason was that the Akt/mTOR signal pathway has been shown to play a crucial role in the induction of key anti-inflammatory and immunomodulatory cytokines [50, 51]. In addition, the activation of the Akt/mTOR signal pathway could be inhibited by oxidative stress [52, 53]. Most importantly, known drugs with greater protective effects against POCD, such as acetylcholinesterase, were found to have the ability to activate the Akt/mTOR pathway [54]. In order to investigate the relationship between the protective effects of edaravone on POCD and activation of the Akt/mTOR pathway, the protein expressions of p-Akt and p-mTOR, as well as SYN intensity, were also tested.

In general, p-Akt participates in regulating cell apoptosis, stimulating cell proliferation, and many other physiological processes [27]. Inflammatory factors such as TNF-a, IL-6, and oxidative factors can inhibit the activation of the Akt/mTOR signal pathway via downregulating the expression of p-Akt protein [55, 56]. mTOR, the main downstream signaling factor in the Akt/mTOR signal pathway, was proven to have a close correlation with cognitive dysfunction such as in Alzheimer’s disease [57]. Moreover, it has also been demonstrated to partially influence synaptic plasticity and memory [24, 25] through regulating the synthesis of certain protein-associated with reshaping of the synapse [58, 59]. Synaptic plasticity was proven to be the biological basis for maintaining learning and memory under normal conditions [60], and SYN-1 intensity was regarded to be involved in regulating the number of synaptic vesicles and contributed to the synaptic function. In the S-P group, the downregulation of expressions of p-Akt and p-mTOR proteins, accompanied by a reduction in SYN intensity in the hippocampi and prefrontal cortex, was observed in the rats; these effects were largely reversed by edaravone, indicating that edaravone could also maintain normal activation of the Akt/mTOR signal pathway by preventing the downregulation of p-Akt and p-mTOR proteins. As a result, neuroinflammation caused by surgery was largely inhibited and synaptic plasticity was maintained, which finally led to the significant attenuation of POCD induced by an operation plus LPS injection.

## 5. Conclusions

In summary, obvious cognitive impairment was shown in rats that underwent a unilateral nephrectomy plus LPS administration. The known antioxidant edaravone could effectively attenuate cognitive impairment; its protective mechanism may be related to its antioxidant and anti-inflammatory effects, as well as its ability to maintain activation of the Akt/mTOR signaling pathway. Although the details of how edaravone improves cognitive function are not yet clear, this paper may provide a new strategy to counter POCD caused by operations.

## Supporting Information

S1 File **Table A.** Average escape latency(s) in the spatial learning of the MWM. **Table B.** MWM test index on day 3 after surgery. **Table C.** Fear conditioning test index. **Table D.** SOD activity (U/mg protein) and MDA concentration (nmol/mg protein) on postoperative day 3. **Table E.** Data5 Number of Iba1-positive cells on postoperative day 3. **Table F.** Ratio of related protein on day 3 after surgery. **Table G.** Density of synaptic protein on postoperative day 3.
(DOC)
