# Supplementary material for: Protective Effects of Edaravone in Adult Rats with Surgery and Lipopolysaccharide Administration-Induced Cognitive Function Impairment
Source: PLoS One. 2016 Apr 26;11(4):e0153708. doi: 10.1371/journal.pone.0153708 (PMC4846078; doi:10.1371/journal.pone.0153708)
Supplement: S1 File — Table A. Average escape latency(s) in the spatial learning of the MWM. Table B. MWM test index on day 3 after surgery. Table C. Fear conditioning test index. Table D. SOD activity (U/mg protein) and MDA concentration (nmol/mg protein) on postoperative day 3. Table E. Data5 Number of Iba1-positive cells on postoperative day 3. Table F. Ratio of related protein on day 3 after surgery. Table G. Density of synaptic protein on postoperative day 3. (DOC) [file pone.0153708.s001.doc]

**Table A. Average escape latency(s) in the spatial learning of the MWM**

| Training day | Day1 | Day 2 | Day 3 | Day 4 | Day 5 |
| --- | --- | --- | --- | --- | --- |
| Mean | 43.06 | 32.95 | 20.40 | 15.47 | 11.66 |
| Std. Deviation | 2.517 | 4.755 | 3.392 | 1.167 | 1.529 |
| Std. Error | 1.258 | 2.377 | 1.696 | 0.5837 | 0.7646 |

**Table B. MWM test index on day 3 after surgery**

| group | C-P | C-E | S-P | S-E |
| --- | --- | --- | --- | --- |
| dwelling time in the target quadrant(s) | 28.14±6.05 | 27.32±6.19 | 20.57±4.69 | 28.68±6.78 |
| the number of crossings | 3.2±1.62 | 2.8±1.81 | 2.22±0.97 | 2.71±1.70 |
| escape latency (s) | 7.59±3.35 | 8.99±7.37 | 28.83±19.46 | 13.32±9.04 |
| swimming speed (m/s) | 0.22±0.07 | 0.25±0.67 | 0.25±0.09 | 0.22±0.09 |

**Table C. Fear conditioning test** index

| group | C-P | C-E | S-P | S-E |
| --- | --- | --- | --- | --- |
| hippocampal-dependent memory test on day 3 after surgery(%Freezing) | 73.78±20.44 | 72.30±21.91 | 39.95±16.49 | 66.58±22.08 |
| hippocampal-dependent memory test on day 7 after surgery(%Freezing) | 75.90±16.63 | 77.53±19.07 | 54.65±12.00 | 76.26±15.71 |
| hippocampal-independent memory test day 3 after surgery(%Freezing) | 73.16±22.58 | 71.87±20.01 | 44.31±13.07 | 71.04±26.15 |
| hippocampal-independent memory test on day 7 after surgery(%Freezing) | 76.35±14.14 | 82.94±12.69 | 70.65±20.35 | 82.10±19.45 |

**Table D. SOD activity (U/mg protein) and MDA concentration (nmol/mg protein) on postoperative day 3**

| group | C-P | C-E | S-P | S-E |
| --- | --- | --- | --- | --- |
| SOD activities in the hippocampus | 102.8±38.31 | 80.84±35.52 | 50.78±20.34 | 71.37±20.23 |
| SOD activities in the prefrontal cortex | 129.7±44.73 | 90.23±55.52 | 54.65±12.00 | 76.26±15.71 |
| MDA level in the hippocampus | 1.56±1.21 | 1.80±0.9974 | 3.65±1.25 | 1.89±0.83 |
| MDA level in the prefrontal cortex | 1.84±1.02 | 1.55±2.00 | 3.30±1.04 | 2.47±0.86 |

**Table E. Data5 Number of Iba1-positive cells on postoperative day 3**

| group | C-P | C-E | S-P | S-E |
| --- | --- | --- | --- | --- |
| hippocampus | 7.00±1.01 | 7.33±0.58 | 14.67±1.53 | 10.02±1.00 |
| prefrontal cortical | 7.12±1.00 | 7.68±1.53 | 16.67±3.22 | 10.33±1.53 |

**Table F. Ratio of related protein on day 3 after surgery**

| group | C-P | C-E | S-P | S-E |
| --- | --- | --- | --- | --- |
| p-Akt/Akt in the hippocampus | 0.73±0.04 | 0.75±0.03 | 0.42±0.05 | 0.55±0.05 |
| p-mTOR/actin in the hippocampus | 0.71±0.04 | 0.75±0.05 | 0.49±0.03 | 0.59±0.03 |
| p-Akt/Akt in the prefrontal cortex | 0.97±0.18 | 0.96±0.31 | 0.51±0.03 | 0.88±0.09 |
| p-mTOR/actin in the prefrontal cortex | 0.48±0.04 | 0.44±0.06 | 0.19±0.14 | 0.38±0.01 |

**Table G. Density of synaptic protein on postoperative day 3**

| group | C-P | C-E | S-P | S-E |
| --- | --- | --- | --- | --- |
| hippocampus | 1.68±0.11 | 1.67±0.088 | 0.74±0.13 | 1.28±0.07 |
| prefrontal cortical | 1.67±0.09 | 1.64±0.06 | 1.52±0.09 | 1.56±0.09 |
